# One-Lung Ventilation via End Tracheostomy for Thoracic Surgery: A Case Report

**DOI:** 10.7759/cureus.77539

**Published:** 2025-01-16

**Authors:** Bridget Ng, Suhitharan Thangavelautham, Maria Aman, Shivani Harikrishnan, Harikrishnan Kothandan

**Affiliations:** 1 Anesthesiology and Perioperative Medicine, Singapore General Hospital, Singapore, SGP; 2 Anesthesiology, Kings Mill Hospital, Nottingham, GBR

**Keywords:** bronchial blocker, double lumen tube, end tracheostomy, one lung ventilation, video-assisted thoracic surgery

## Abstract

Standard double-lumen tubes (DLTs) are unsuitable in patients with end tracheostomy due to the shortened tracheal length and atypical stoma angulation. This case highlights the challenges of achieving one-lung ventilation (OLV) in patients with end tracheostomies due to anatomical changes following total laryngectomy. A 68-year-old male with a history of total pharyngo-laryngo-esophagectomy, gastric pull-up, and end tracheostomy underwent video-assisted thoracic surgery (VATS) for left lung wedge resection and lymph node dissection. A #7.5 Portex cuffed tracheostomy tube and a Coopdech bronchial blocker (BB) were used to achieve OLV. The Coopdech BB was placed intraluminally through the tracheostomy tube into the left main bronchus, and the lung isolation was confirmed clinically via auscultation and subsequently with the guidance of fiberoptic bronchoscopy. BBs are often preferred for their flexibility and ability to accommodate tracheostomy anatomy while minimizing stoma trauma. This approach provided stable lung isolation and accommodated anatomical variations. The case underscores the importance of tailoring OLV strategies to patient-specific anatomical challenges in patients with end tracheostomy.

## Introduction

Total laryngectomy, a surgical procedure involving the complete removal of the larynx, permanently alters airway anatomy by separating the trachea from the pharynx and creating an end tracheostomy. This anatomical modification poses unique challenges for airway management, particularly in achieving one-lung ventilation (OLV). OLV is essential during VATS to provide a clear surgical field by collapsing the lung on the operative side while maintaining adequate oxygenation and ventilation on the non-operative side. OLV is typically achieved using double-lumen endotracheal tubes (DLTs), allowing independent lung ventilation. Key factors influencing airway selection include the size of the stoma, the anatomy of the trachea, and the presence of airway abnormalities. Placing a DLT in patients with end tracheostomies is challenging as it requires precise airway manipulation, taking into account tracheostomy stoma size, airway abnormalities, a small or restricted stoma, and because a total laryngectomy will limit oral-tracheal intubation [1]. Standard DLTs were unsuitable due to shortened tracheal length and atypical stoma angulation [2].

This case report describes a successful approach to OLV in a patient with a history of total pharyngo-laryngo-esophagectomy, gastric pull-up, and end tracheostomy undergoing video-assisted thoracic surgery (VATS) for left lung wedge resection and lymph node dissection. Instead of a DLT, OLV was achieved using a cuffed tracheostomy tube and a bronchial blocker (BB), demonstrating an alternative strategy tailored to the unique airway challenges in such patients. This report highlights the clinical significance of selecting appropriate devices and techniques for OLV in individuals with end tracheostomies, offering insights into their advantages, limitations, and implications for surgical and anesthetic management.

## Case presentation

A 68-year-old male presented with metachronous squamous cell carcinoma involving the upper and lower lobes of the left lung. The patient was scheduled for elective VATS, wedge resection of the left upper and lower lobe, and lymph node dissection. His medical history included hypertension, diabetes mellitus, ischemic heart disease, hypothyroidism, and a prior diagnosis of cervical esophageal squamous cell carcinoma which was treated in 2013 with total pharyngo-laryngo-esophagectomy, gastric pull-up, and an end tracheostomy.

**Figure 1:**
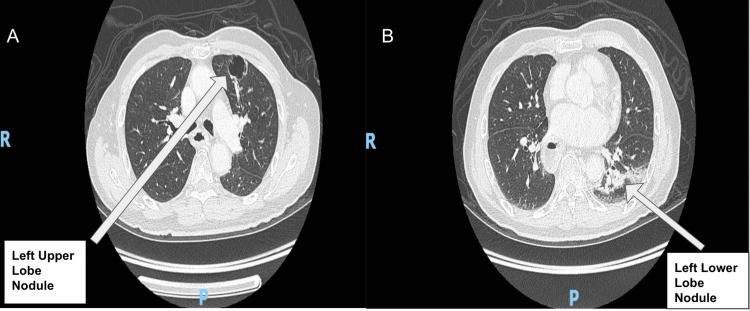
Computed tomography (CT) scan showing a 2.2 cm nodule in the left upper lobe and a 1.4 cm nodule in the left lower lobe.

**Figure 2:**
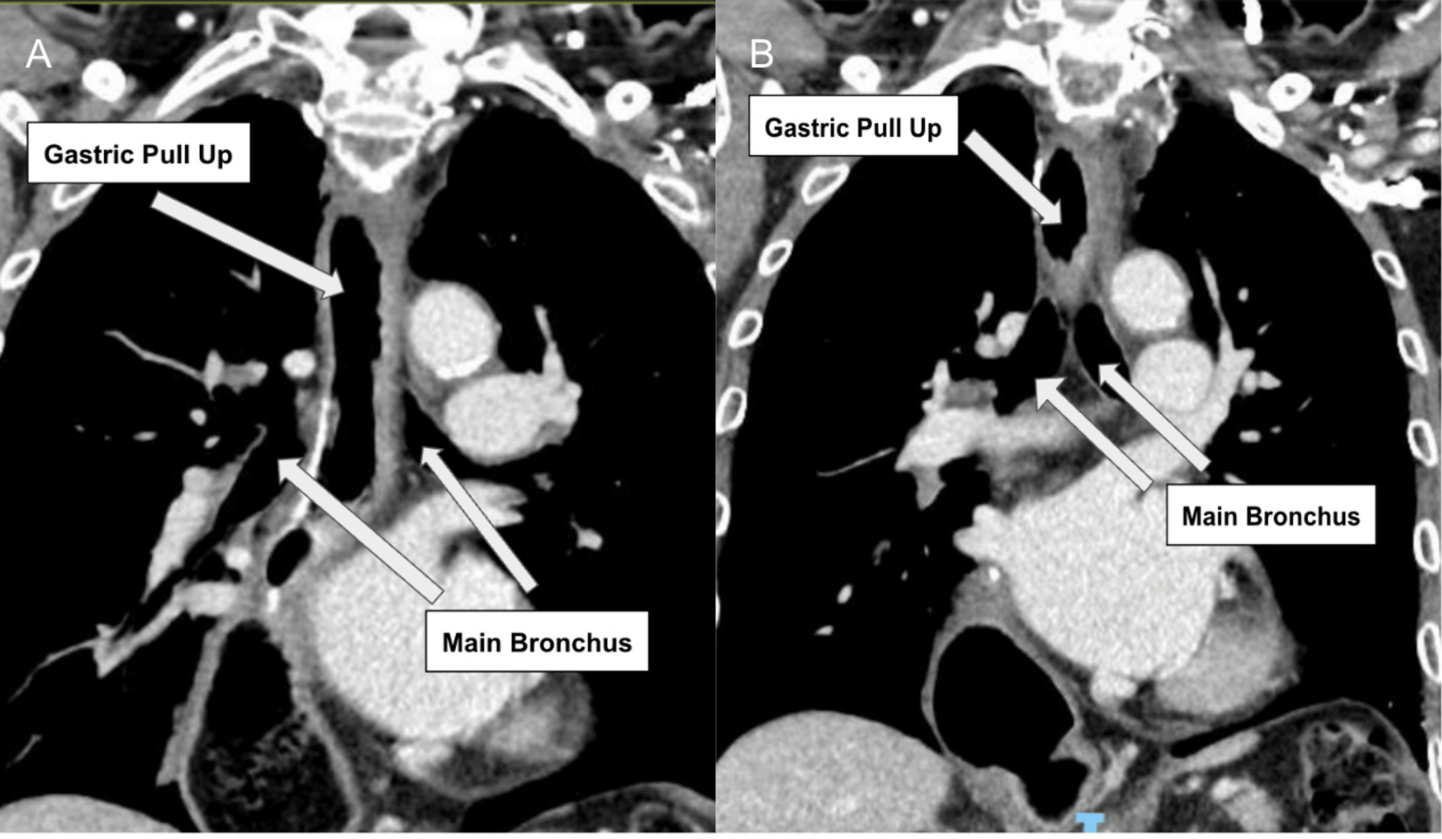
Coronal view of a computed tomography scan of the thorax in a patient with a previous total pharyngo-laryngo-esophagectomy, showing the gastric pull-up and the two main bronchi.

**Figure 3:**
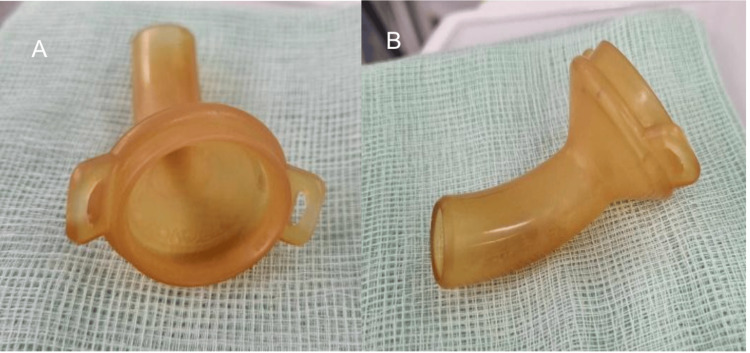
The patient's laryngostomy tube reveals a patent tracheostomy without obstruction upon removal.

The diagnosis of metachronous squamous cell carcinoma was made following routine surveillance imaging, which detected lung nodules during cancer follow-up. The patient was asymptomatic and had a good functional status. Preoperative imaging revealed a 2.2 cm nodule in the left upper lobe margin and a 1.4 cm nodule in the left lower lobe (Figure 1). A computed tomography (CT) scan of the thorax revealed the two main bronchi with gastric pull-up (Figure 2). The laryngectomy opening diameter was 13.8 mm. Preoperatively, the patient had no difficulty breathing, shortness of breath, or orthopnea and could walk for one hour with good functional status. He used a laryngostomy tube, which revealed a patent tracheostomy without obstruction once removed (Figure 3). 

Following extensive preoperative discussion, various OLV strategies were considered, including inserting a tracheostomy tube with a BB, inserting a single-lumen tube (SLT) with a BB, and endobronchial SLT insertion. A preoperative discussion was conducted with both the patient and surgeons, and it was agreed that if lung isolation was not possible with the above options, the procedure would be canceled and alternative treatments could be considered. Standard airway equipment was prepared, including Portex tracheostomy tubes of sizes 6.5, 7.0, and 7.5, SLTs of sizes 6.0 and 7.0, DLTs (35 French and 37 French), and a Coopdech BB. 

The patient was pre-oxygenated with 100% oxygen, and the end tracheostomy stoma was anesthetized with co-phenylcaine spray before the induction of anesthesia. Intravenous (IV) remifentanil 0.02 µg/kg/min was administered to facilitate the exchange of the laryngostomy tube for a size 7.5 Portex cuffed tracheostomy tube. The tracheostomy tube was then connected to the anesthesia circuit. The patient was then induced with propofol 90 mg and rocuronium 50 mg. Sevoflurane at a concentration of 2.0% to 2.2% was used to maintain anesthesia. Lung isolation was achieved using a Coopdech BB, placed intraluminally through the tracheostomy tube into the left main bronchus. The isolation was confirmed clinically via auscultation and subsequently with fiberoptic bronchoscopy using an Olympus 3.8 mm scope. The patient was positioned in the right lateral decubitus position for the surgery, and the lung isolation was reconfirmed. 

Anesthesia was maintained with remifentanil, sevoflurane, and oxygen and air mixture at 2 L/min. During the operation, muscle relaxation was maintained by administration of intravenous rocuronium. The total anesthetic duration was three hours, and the duration of OLV was two hours and 25 minutes. In the lateral position, the peak airway pressure (PAP) of two lung ventilation was 21 cmH2O. During OLV, we tried to keep the PAP at or under 20 cmH2O. Volume-controlled ventilation was set with the following settings: FiO2 0.4, tidal volume (TV) 450 mL, positive end-expiratory pressure (PEEP) 5 mmHg, and a respiratory rate (RR) of 11 breaths per minute. Vital signs during the surgery were within normal range. After the reversal of the muscle relaxant and the return of spontaneous respiration with adequate TV, the BB was removed, and the tracheostomy tube was replaced with the laryngostomy tube. The patient was transferred to recovery with supplemental oxygen delivered via a tracheostomy mask.

## Discussion

Overview of OLV and its techniques 

OLV is essential during VATS to provide a clear surgical field by collapsing the lung on the operative side while maintaining adequate oxygenation and ventilation on the non-operative side. There are commonly three different methods that can be used to achieve lung isolation and OLV: DLTs, BBs, and SLTs advanced into either the right or left main-stem bronchus. DLTs are the most commonly used device for achieving OLV, allowing the selective ventilation of one lung while the other is collapsed to provide a better surgical field. BBs are an alternative to DLTs and are particularly useful in patients with difficult airways, standard tracheostomies, or when DLT placement is challenging. SLT main stem intubation is typically used in emergent settings. Provided are tables comparing the external and internal diameters of various sizes of Mallinckrodt (Bronch Cath) DLTs (Table 1), SLTs with BBs (Table 2), and the sizes of Ambu flexible fiberoptic bronchoscopes (Table 3) available at our institution. All data were obtained from the manufacturer's catalog. 

**Table 1:** Comparison of outer and inner diameters of Mallinckrodt double-lumen tubes (DLTs).

DLT size (French)	Internal diameter (mm)	Outer diameter (mm)
28	3.1	9.3
32	3.4	10.7
35	4.8	11.7
37	5.1	12.3
39	5.3	13.0
41	5.4	13.7

**Table 2:** Comparison of outer and inner diameters between single-lumen tubes (SLTs) and SLTs with bronchial blockers. NA, not applicable or not available

SLT internal diameter (mm)	SLT outer diameter (mm)	SLT with a bronchial blocker (Univent) internal diameter (mm)	SLT with a bronchial blocker (Univent) outer diameter (mm)
5.0	6.8 mm	NA	NA
5.5	7.4 mm	NA	NA
6.0	8.2 mm	6.0	9.7 mm
6.5	8.8 mm	6.5	10.2 mm
7.0	9.6 mm	7.0	10.7 mm
7.5	10.2 mm	7.5	11.2 mm
8.0	11 mm	8.0	11.7 mm

**Table 3:** Ambu Scope outer diameter and distal end diameter.

Type of Ambu Scope	Outer diameter (mm)	Distal end diameter (mm)
Slim	3.8	4.2
Regular	5.0	5.4
Large	5.8	6.2

Management challenges of OLV in patients with end tracheostomy

Managing OLV in patients with an end tracheostomy for procedures like VATS presents unique challenges compared to standard tracheostomies. After a total laryngectomy (e.g., pharyngo-laryngo-esophagectomy with gastric pull-up), the trachea is surgically separated from the pharynx, creating a permanent end tracheal stoma. This complete anatomical separation contrasts with a standard tracheostomy, where a patent airway above the stoma allows for potential transoral intubation if complications arise. Various OLV strategies are available for patients with end-tracheostomies such as cuffed tracheostomy tubes with BBs, SLTs combined with BBs, and endobronchial intubation with an SLT. Optimal device selection depends on factors such as the tracheostomy's age, diameter, stoma angle, and the presence of airway abnormalities [1,3]. Each of these variables helps in selecting appropriate equipment and in anticipating challenges for achieving effective lung isolation.

Cuffed tracheostomy tube with BB

In our patient, we utilized a cuffed tracheostomy tube with a Coopdech BB. Campos et al. previously reported cases describing various methods of OLV in patients with tracheostomy, using a BB through a tracheostomy cannula or a single-lumen endotracheal tube [1]. Using these techniques, they were able to achieve effective lung isolation in patients with long-term tracheostomies, while also enabling continuous oxygen administration. In our patient, we utilized a tracheostomy tube as the length of the tracheostomy tube is shorter compared to an SLT, with a reduced chance of kinking and trauma [4]. It is also easier to secure as compared to the SLT. Tracheostomy tubes are also designed specifically for the stoma and provide a more stable fit, reducing the risk of tube displacement or movement, which can be more common with SLT. 

Endotracheal SLT with BB

Alternatively, OLV for patients with end tracheostomy may be achieved using an SLT combined with a BB. BBs, such as the Arndt and Uniblocker, are advantageous because they can be used with either a tracheostomy tube or a SLT, allowing for intraluminal or extraluminal placement. Lim et al. [5] reported a clinical experience of OLV in a patient using an endobronchial blocker with a single-lumen reinforced wire endotracheal tube with permanent tracheostomy after total laryngectomy. Similarly, Seo et al. [6] reported on the clinical experience of OLV anesthesia using a Univent® tube (Fuji Systems Corporation, Japan) for a patient who had undergone a total laryngectomy.

Although DLTs are often preferred for OLV and lung isolation since they allow the clearing of blood or respiratory secretions from the operated lung, a BB is mostly preferred for OLV in patients with tracheostomy. A BB’s smaller external diameter and greater flexibility facilitate easier securement of blockers and reduce the risk of injury to the surrounding tissue of the permanent tracheostomy stoma compared to a DLT. The extraluminal placement of BBs, as described by Sekhon et al. [7], offers specific advantages, like maintaining a smaller, unobstructed SLT lumen, facilitating suctioning and bronchoscopy, and reducing the risk of migration. However, BBs may also pose limitations, such as malposition risk, limited suction capability, slower lung collapse, and challenges in transitioning between one- and two-lung ventilation [8]. 

DLT placement 

The most common method of lung isolation is the placement of a DLT. However, this has unique challenges for our patient with an end tracheostomy. The shorter length of the trachea results in a greater length of the extra tracheal part of the tube, which can lead to some difficulties in fixing and keeping the tube in the correct position. End tracheostomies also complicate the insertion and alignment of DLTs, as anatomical alignment at the stoma differs from standard orotracheal intubation [9]. The altered angle at the stoma can make DLT insertion difficult and increase the risk of airway trauma and the creation of a false tract during tube exchange, particularly in patients with recent tracheostomies. However, there have been case reports of successful insertion of a DLT through a tracheostomy. Coe et al. [10] reported on eight cases in which a DLT was inserted through the stoma of a tracheostomy, with successful OLV.

Alternative methods for OLV 

Other methods for OLV in an end tracheostomy include the usage of an SLT-positioned endobronchial to achieve OLV. Armored SLTs, for instance, have a reduced risk of kinking and offer improved maneuverability. However, the disadvantage of this approach includes limited suctioning of the contralateral lung and slower deflation. Furthermore, alternative methods for OLV in patients with tracheostomy also include a laryngeal mask airway (LMA) combined with a BB, as described by Robinson et al. [11]. However, this is effective only if the upper airway anatomy allows for LMA placement, and this is not feasible in our patient who had an end tracheostomy.

## Conclusions

The perioperative management of OLV in patients with an end tracheostomy undergoing thoracic surgery requires careful planning, tailored strategies, and multidisciplinary collaboration. This case demonstrated the effective use of a Coopdech BB with a cuffed tracheostomy tube, providing stability, precise lung isolation, and minimal airway trauma. While alternative methods like DLTs and SLTs with BBs are viable, the choice of technique should consider patient-specific factors, such as stoma size and airway anatomy. The experience highlights BBs with cuffed tracheostomy tubes as a versatile, safe, and reliable option for managing complex airway cases.
